# Evaluation of the Antibacterial Properties of Turmeric-, Synephrine- and Yohimbine-Containing Dietary Supplements Used by Bodybuilders

**DOI:** 10.3390/microorganisms14102399

**Published:** 2026-10-20

**Authors:** Nataliia Tkachuk, Liubov Zelena, Yuliia Denysenko, Dmytro Nikolaienko, Svitlana Zahorodnia

**Affiliations:** 1 Department of Biology and Human Health, T.H. Shevchenko National University “Chernihiv Colehium”, 14013 Chernihiv, Ukraine; y.denysenko@chnpu.edu.ua (Y.D.); dm.nikolaenko@chnpu.edu.ua (D.N.); 2 Department of Virus Reproduction, D.K. Zabolotny Institute of Microbiology and Virology, NAS of Ukraine, 03680 Kyiv, Ukraine; zelena@nas.gov.ua (L.Z.); s.zagorodnya@imv.org.ua (S.Z.)

**Keywords:** antibacterial assay, antibacterial properties, ergogenic agents, *Escherichia coli*, health, plant-based preparations, sport, *Staphylococcus aureus*

## Abstract

Among the substances and preparations used in bodybuilding as potential or auxiliary ergogenic agents, particular attention should be paid to plant-based, specifically turmeric, synephrine and yohimbine, preparations due to their known antimicrobial properties. Despite the known antibacterial properties of individual active components, the antimicrobial activity of commercial ergogenic foods and sports supplements formulated with them remains insufficiently studied. Therefore, the aim of this study was to study the in vitro antibacterial activity of commercial dietary supplements with turmeric, synephrine and yohimbine used by bodybuilders against some representatives of sanitary indicator bacteria—*Escherichia coli* and *Staphylococcus aureus*. The study used commercial dietary supplements freely available in Ukraine, which, according to the manufacturers, contained turmeric, *Citrus aurantium* extract (with synephrine) and yohimbine. Gram-negative *E. coli* ATCC 35218 and Gram-positive *S. aureus* ATCC 29213 were used as model test systems. Antibacterial susceptibility was assessed by a spot-on-lawn antibacterial assay (on MPA) and a method with a liquid nutrient medium (in MPB), with subsequent statistical processing of the results. The preliminary results obtained demonstrate moderate antibacterial activity of the studied food supplements used by athletes, primarily turmeric- and synephrine-containing supplements, against the studied test bacterial cultures. Given the results obtained, it is advisable to conduct further research on dietary supplements containing turmeric and synephrine to evaluate their antibacterial potential, which requires further study, particularly involving a broader range of test strains. Future studies should focus on specific molecular mechanisms of action, establishing effective concentrations, and assessing safety during long-term use. Particular attention should be paid to potential interactions with commonly used antibacterial drugs and to the risks of antibiotic resistance.

## 1. Introduction

**Figure 1:**
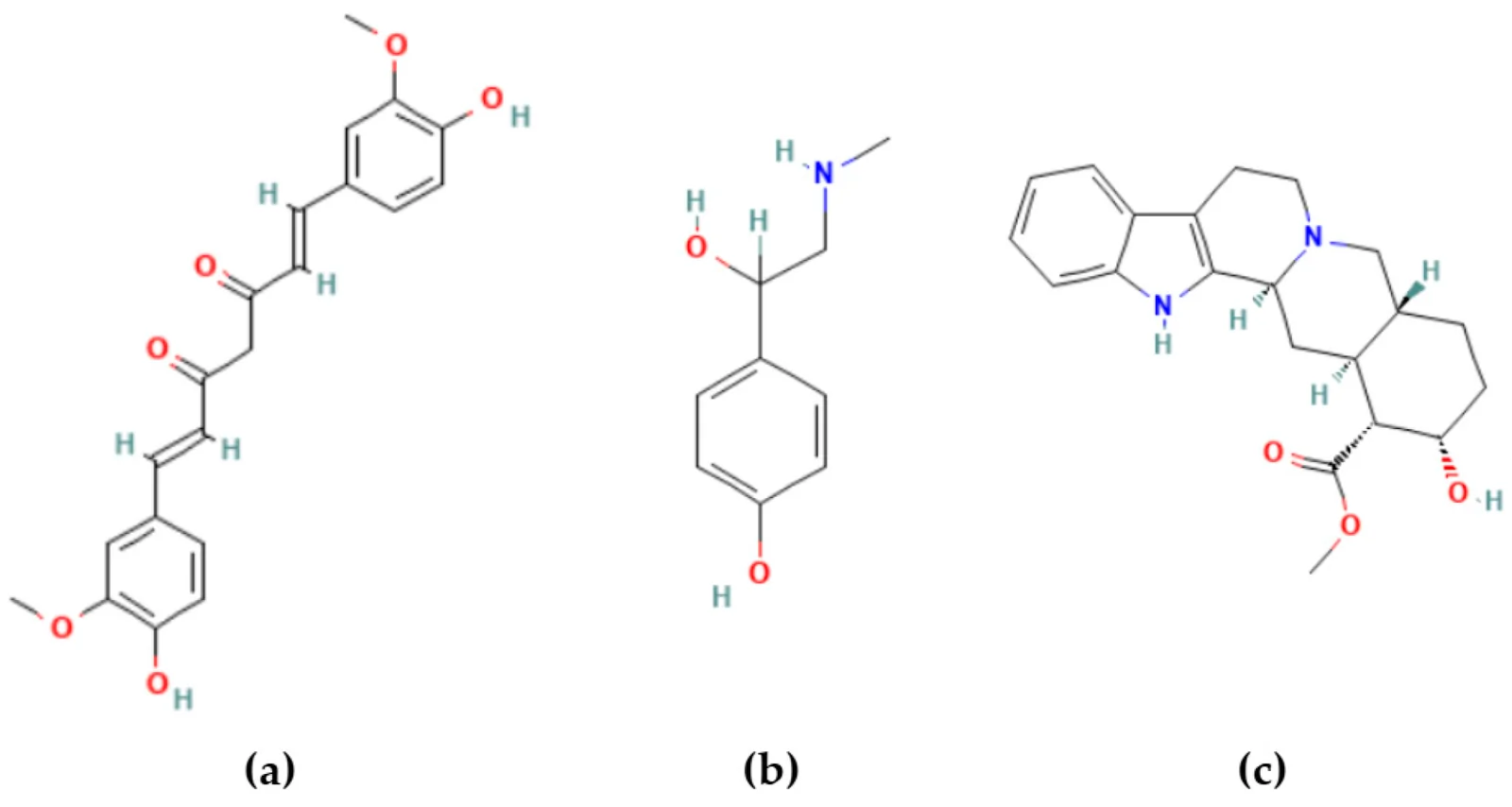
Two-dimensional structures of certain substances in plant-based preparations used in sports practice: (**a**) curcumin [17]; (**b**) synephrine [18]; (**c**) yohimbine [19].

Enhancing endurance and maintaining performance during training depends on many dietary factors, such as the intake of micro- and macronutrients compatible with energy and water balance [1]. Dietary supplements, in particular ergogenic supplements, which are sometimes used by bodybuilders, can increase muscle strength, training performance and improve body composition, especially during periods of preparation for competitions [2]. Among the substances and preparations used in sports practice as potential or auxiliary ergogenic aids, plant-based preparations deserve attention, in particular, those containing: curcumin—the active ingredient in turmeric [3,4]; synephrine—the active ingredient in the fruits of citrus species [5,6]; yohimbine—an ingredient from the bark of the *Corynanthe yohimbe* tree (West Africa) [7], as these active ingredients are also known in the context of their antimicrobial properties. Curcumin (also known as diferuloylmethane)—a beta-diketone (Figure 1a)—helps athletes reduce inflammation and muscle soreness after exercise [8,9]. Some studies also indicate its antimicrobial activity [10,11]. Synephrine (also referred to as *p*-synephrine) (Figure 1b) is a phytoalkaloid from *Citrus aurantium* that exhibits anti-inflammatory and antitumor activity both in vitro and in vivo. It influences α- and β-adrenergic receptors, possesses low toxicity and demonstrates metabolic effects, including lipolysis and thermogenesis [12,13]. Yohimbine is an indole alkaloid (Figure 1c) with broad pharmacological potential that interacts with serotonin (5-HT) and dopamine receptors, stimulates the central nervous system, and exhibits vasoactive effects. It is characterized by potential anti-inflammatory and antitumor effects, and is also promising for the treatment of erectile dysfunction, cardiovascular disorders, and neurological conditions [7,14,15,16].

A number of ergogenic aids have biological properties, in particular, antimicrobial activity [20,21]. Food supplements containing turmeric, synephrine and yohimbine are widely used in sports nutrition, and these biologically active components possess recognized antibacterial properties. However, the antibacterial properties of fully formulated ergogenic foods and sports supplements, which contain various excipients besides active components, remain understudied. Consequently, evaluating the antibacterial activity of complex supplements against key Gram-negative and Gram-positive pathogens, in particular *Escherichia coli* and *Staphylococcus aureus* as primary pathogens threatening human health [22,23], is necessary to characterize their potential biological effects, which will allow characterizing this potential biological effect of complex preparations.

The identification of additional biological effects of drugs—both beneficial and adverse—requires a detailed analysis of their pleiotropic effects, as this provides the basis for substantiating the potential of drug repositioning by defining new therapeutic indications [24]. In particular, a number of well-known drugs (e.g., amlodipine, pentamidine, sertraline, aspirin, flupentixol, and others) have demonstrated new properties—antibacterial or antifungal [25]. Therefore, it is relevant to evaluate the antibacterial activity of nutritional supplements consumed by bodybuilders and containing components with potential antibacterial properties. This study aims to examine the in vitro antibacterial efficacy of commercial turmeric-, synephrine-, and yohimbine-containing supplements preferred by bodybuilders against representatives of sanitary indicator bacteria—*E. coli* and *S. aureus*.

## 2. Materials and Methods

### 2.1. Dietary Supplements Used in the Study

The study used commercial dietary supplements freely available in Ukraine, which, according to the manufacturers, contained turmeric (**Tur**), *C. aurantium* extract (with synephrine) (**Cia**) and yohimbine (**Yoh**). Based on the information on the packaging, the characteristics of the studied dietary supplements (excluding the capsule shell composition, which was not used in the study) are as follows:

• **Tur** (dietary supplement Turmeric Extract Haya Labs, Haya Labs LLC, Washington, DC, USA)—1 capsule contains 500 mg of turmeric extract: 90% curcuminoids—contains curcumin, demethoxycurcumin, bismethoxycurcumin; other ingredients—magnesium stearate.
• **Cia** (Nutrend Synephrine, NUTREND D.S., Olomouc, Czech Republic)—1 capsule contains maltodextrin and 125 mg of *C. aurantium* extract, in which synephrine content is 10 mg.
• **Yoh** (“Zdorovya” Corporation LLC (Kharkiv, Ukraine))—1 tablet contains 5 mg of yohimbine hydrochloride. Excipients: potato starch; lactose, monohydrate; calcium stearate; hypromellose; cellactose (a mixture of lactose, monohydrate, and powdered cellulose (75:25)).

Alcohol solutions at concentrations of 5 mg/mL and 0.5 mg/mL of the active components (turmeric, synephrine, yohimbine) of the dietary supplements were used in further experiments. For the spot-on-lawn antibacterial assay, solutions with a concentration of 5 mg/mL were used. To screen for and assess the antibacterial activity of a compound in a liquid nutrient medium, the solutions were used as stock solutions for the preparation of working solutions, which provided final concentrations of the studied active components (turmeric, synephrine, yohimbine) in the nutrient medium (meat-peptone broth—MPB) of 300 and 30 μg/mL. Ethyl alcohol (96%) was used as a solvent and solvent control in studies of the antibacterial effect of the preparations. The concentrations of active plant components in the dietary supplements were selected to exceed the known levels required to exert an antibacterial effect (curcumin: 187.5 µg/mL for *S. aureus* ATCC 25923 [26]; synephrine: 8 µg/mL for *E. coli* ATCC 35218 and 16 µg/mL for *S. aureus* ATCC 25923 [27]; yohimbine: 4 µg/mL for *E. coli* ATCC 35218 and 2 µg/mL for *S. aureus* ATCC 25923 [27]), thereby ensuring the expected outcome was achieved.

### 2.2. Bacterial Test Cultures

Overnight MPB cultures of Gram-negative *E. coli* ATCC 35218 and Gram-positive *S. aureus* ATCC 29213 served as the test bacterial strains in this study. The initial titer of the bacterial suspensions was 1.5 × 10^8^ colony-forming units (CFU)/mL (0.5 according to McFarland). The suspensions were prepared aseptically, focusing on optical density using the VITEK^®^ DensiCHEK^®^ apparatus (bioMérieux S.A., Marcy l’Étoile, France). Working bacterial suspensions with a concentration of 10^6^ CFU/mL were prepared from the initial suspensions of 1.5 × 10^8^ CFU/mL. The volume of the bacterial suspensions was: for a spot-on-lawn antibacterial assay—1 mL in each Petri dish; for a method with a liquid nutrient medium—0.1 mL in each test tube with the appropriate dilution of the respective dietary supplements or control culture.

### 2.3. Antibacterial Bioassay

#### 2.3.1. Spot-on-Lawn Antibacterial Assay

For the initial screening of the antibacterial properties of the respective dietary supplements, the spot-on-lawn antibacterial assay was used [28,29] with some modifications. In the study, 1 mL of the appropriate bacterial suspension was added to sterile Petri dishes, sterile chilled but still liquid meat-peptone agar was added, mixed and left until the medium solidified. Alcoholic solutions (0.1 mL) of the appropriate active components of the dietary supplement with a concentration of 5 mg/mL were spotted onto the surface of the seeded solidified medium. The plates inoculated with bacteria were incubated in a thermostat at 35 °C for 24 h. After the incubation time, the diameter of the growth inhibition zone around the site of drug application was measured with a ruler. The antibiotic tobramycin at a concentration of 10 μg/disk served as a positive control (Figure S1).

#### 2.3.2. Evaluation of Antibacterial Activity of the Food Supplements in a Liquid Nutrient Medium

**Figure 2:**
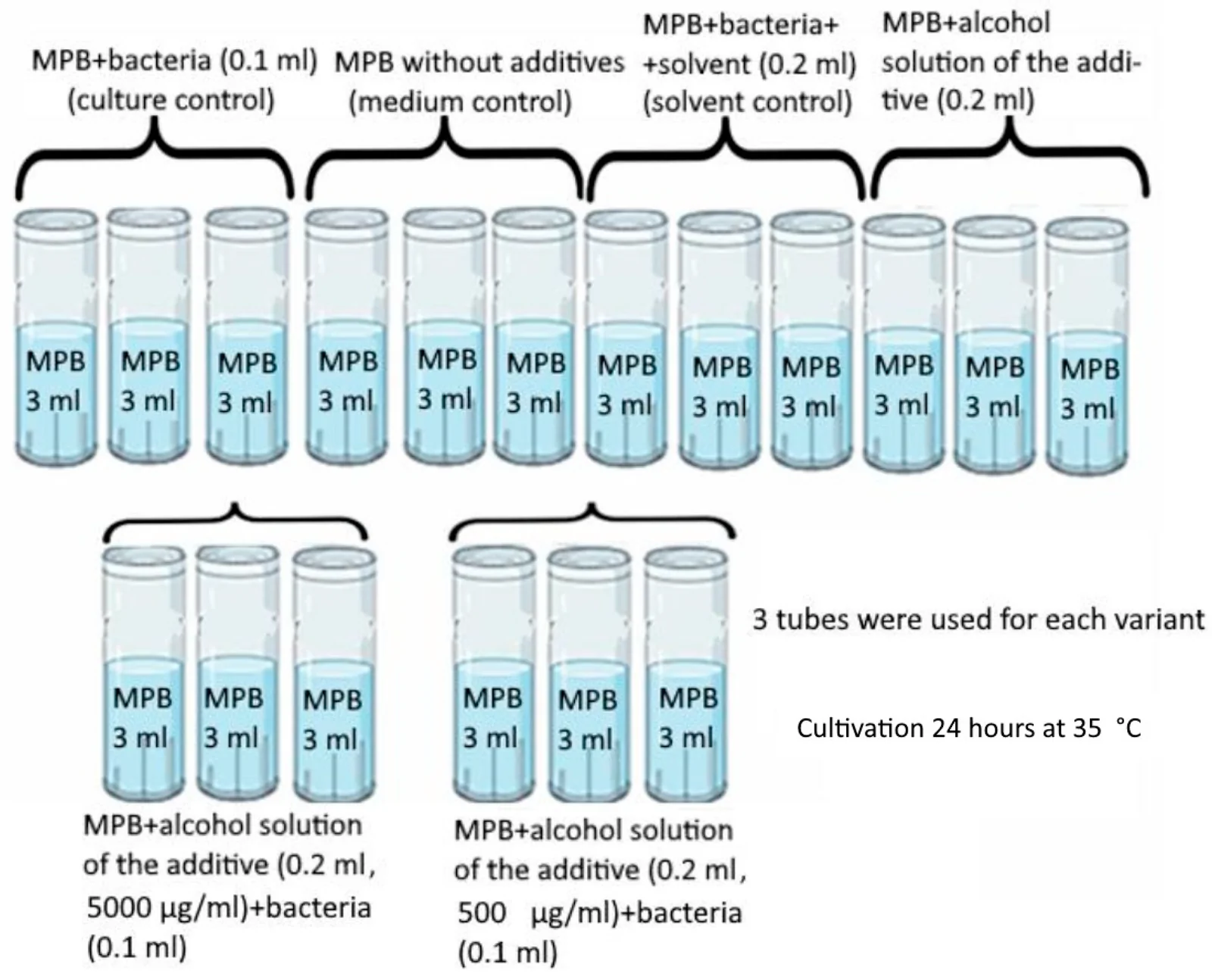
Scheme of the experiment on the antibacterial properties of dietary supplements in the liquid nutrient medium.

The scheme of the method for assessing the antibacterial properties of food supplements is shown in Figure 2.

The final concentration of the respective active substance of each dietary supplement in MPB was 300 and 30 μg/mL in the medium. The tubes were incubated in a thermostat at 35 °C for 24 h, followed by measurement of the optical density of the tube contents using the VITEK^®^ DensiCHEK^®^ apparatus (bioMérieux S.A., Marcy l’Étoile, France), with the results expressed in McFarland units (McFarland, McF). Controls used in the experiment: culture control (with the addition of the respective bacterial culture without supplements); sterility control (sterile medium without supplements); sterility controls of the alcoholic solution of the dietary supplements (sterile medium with an alcoholic solution of the respective dietary supplement—Figures S2 and S3); positive control (the drug “Tobrex” (Novartis Manufacturing NV, Puurs, Belgium) containing tobramycin at a concentration of 3 mg/mL with excipients benzalkonium chloride, boric acid, sodium sulfate anhydrous, sodium chloride, tyloxapol, sulfuric acid and/or sodium hydroxide, purified water, from which working solutions were prepared with a final antibiotic concentration in the nutrient medium of 300 and 30 μg/mL).

### 2.4. Statistical Processing of Results

The study was conducted in triplicate. For statistical processing of the data obtained in the experiment, we used Microsoft Excel to calculate the arithmetic mean (M) and the standard error of the mean (SEM, m) and the statistics software package PAST (version 4.03) [30] for scientific data analysis. Data were analyzed using one-way analysis of variance (one-way ANOVA) followed by Tukey’s HSD post-hoc test to determine differences among treatments.

## 3. Results and Discussion

**Figure 3:**
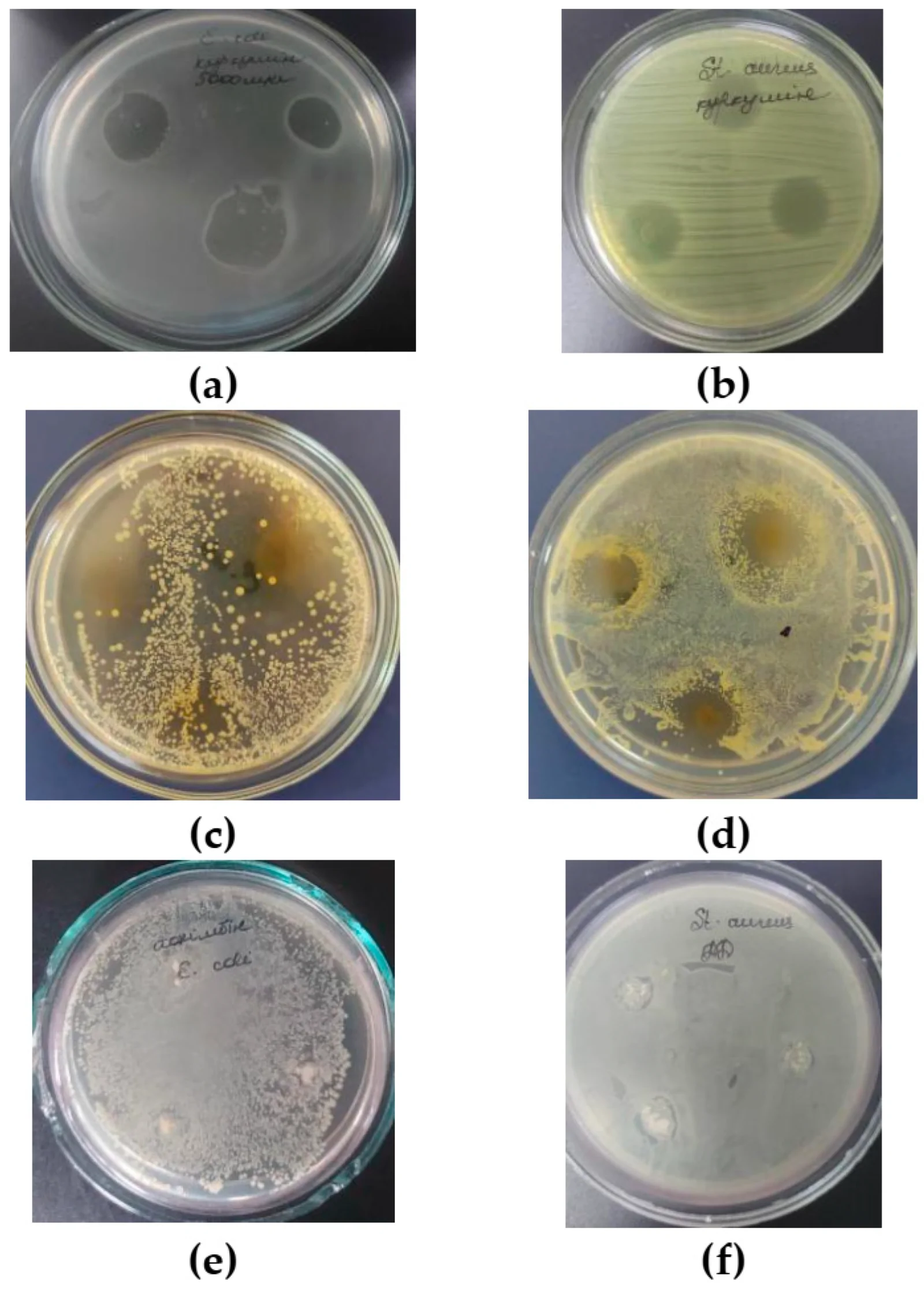
Antibacterial effect of the food supplements (spot-on-lawn antibacterial assay): (**a**) *E. coli* (**Tur** with turmeric); (**b**) *S. aureus* (**Tur** with turmeric); (**c**) *E. coli* (**Cia** with *C. aurantium* extract containing synephrine); (**d**) *S. aureus* (**Cia** with *C. aurantium* extract containing synephrine); (**e**) *E. coli* (**Yoh** with yohimbine); (**f**) *S. aureus* (**Yoh** with yohimbine).

The results of the study of the antibacterial properties of food supplements are presented in Figure 3, Figures S1–S3 and in Table 1 and Table 2.

Bacterial test cultures were found to be moderately sensitive to **Tur** at a turmeric concentration of 5 mg/mL (diameters of inhibition zone were equal to 18.0 ± 3.0 mm for *E. coli* and 18.3 ± 0.3 mm for *S. aureus*) (Figure 3, Table 1), compared with tobramycin (27.2 ± 0.7 mm and 35.0 ± 2.9 mm, respectively) (Figure S1).

The results of the liquid medium assay at **Tur** concentrations of 300 and 30 μg turmeric/mL showed a two- to threefold decrease in the optical density of the analyzed samples (Figure S2, Table 2). In comparison, treatment with tobramycin (positive control) led to no bacterial growth after subculturing, confirming its bactericidal effect (Figure S2, Table 2).

Therefore, the obtained data indicate the susceptibility of bacterial test cultures to the dietary supplement containing turmeric—**Tur** (a dietary supplement recommended for supporting the immune system and bone health [31]), which confirms the previously mentioned antibacterial property of its active components—curcumin [32,33,34,35,36], demethoxycurcumin [37,38] and bismethoxycurcumin [39,40]. More specifically, the inhibitory effect of turmeric leaf extract (at concentrations of 20%, 40%, 60%, and 80%) on the growth of *E. coli*, *S. aureus*, and *Shigella dysenteriae*, as well as the absence of an inhibitory effect on the growth of *Lactobacillus acidophilus*, was previously noted [41]. However, there is a report of the absence of a zone of growth inhibition for any of the bacterial isolates associated with urinary tract infection, as well as the standard ATCC strains of *E. coli* and *S. aureus,* against all the turmeric extracts (20%, *w*/*v*) in distilled water, ethanol and methanol [42]. Curcumin can modulate the balance between beneficial microbiota and pathogens, as evidenced by a reduction in pathogenic bacterial proliferation upon exposure [32,33,34,35,36].

Among the antibacterial mechanisms of action of curcumin, the formation of antioxidant oxidation products, inhibition of bacterial virulence factors, inhibition of bacterial biofilm formation, and prevention of bacterial adhesion to host receptors through the bacterial quorum sensing regulatory system are considered [43]. It may also exhibit synergistic antibacterial effects in combination with other antibacterial agents [43]. In addition to curcumin, the composition of the studied dietary supplement included (according to the manufacturer) other active ingredients of turmeric: demethoxycurcumin, which is an analogue of curcumin chemically, without a methoxy group directly linked to the benzene ring, which makes this structure more stable in activity and solubility in water than curcumin at physiological pH [38], bismethoxycurcumin—a beta-diketone that is methane in which two of the hydrogens are substituted by 4-hydroxycinnamoyl groups [44] and magnesium stearate as auxiliary compounds—an organic magnesium salt [45]. Demethoxycurcumin exhibits a variety of biological activities: anti-inflammatory, antihypertensive, neuroprotective, antimalarial, and vasodilator, as well as antifungal and antimicrobial effects [37,38]. The antibacterial mechanism of demethoxycurcumin is through the inhibition of transpeptidase, which prevents the synthesis of glycopeptides in the bacterial cell wall. Demethoxycurcumin can modulate cell proliferation and inflammatory signaling pathways [46]. Bismethoxycurcumin also demonstrated potential antibacterial properties and synergistic effects when combined with antibiotics, particularly against methicillin-resistant *S. aureus* [39]. According to Wang et al. [40], subinhibitory concentrations of bismethoxycurcumin significantly suppressed the expression of genes of *Staphylococcus* enterotoxin-related exoproteins (enterotoxin A, enterotoxin B) and α-toxin: *sea*, *seb* and *hla* at the mRNA level in methicillin-resistant *S. aureus*. Suppression of the accessory regulatory gene *agr* caused a decrease in the expression of virulence-related exoproteins and inhibited biofilm formation following treatment with the compound at subinhibitory concentrations. These observations suggest that bismethoxycurcumin has potential as a natural antibacterial agent to address the growing threat of antibiotic resistance [40].

However, for such an additive component as magnesium stearate, antibacterial properties against *Salmonella typhimurium* strains TA100 and TA1535 and *E. coli* strain WP2uvr A were not detected [47].

Among the studied bacteria, *E. coli* were found to be more sensitive to the effects of the alcoholic solution of the food additive **Cia** (Figure 3, Table 1).

According to the manufacturer, this supplement used for body shaping, weight control and thermogenesis support contains maltodextrin and synephrine-rich *C. aurantium*.

At a concentration of *C. aurantium* extract of 62.5 mg (synephrine 5 mg)/mL in the alcoholic solution of **Cia**, the diameter of bacterial growth inhibition zones was 39.7 ± 2.6 mm for *E. coli* and 18.0 ± 0.6 mm for *S. aureus* (Figure 3, Table 1). Thus, the food additive with synephrine-containing *C. aurantium* extract demonstrated antibacterial activity against the studied bacterial test cultures when using the agar medium method. This is consistent with the data on the antibacterial activity of *C. aurantium* extract components using agar medium [48,49]. However, it was not confirmed by the method using a liquid medium (meat-peptone broth): the optical density of control bacterial culture and treated with food additive **Cia** (with concentrations of *C. aurantium* extract 375 and 3750 μg (synephrine 30 and 300 μg)/mL of medium) did not differ significantly (Figure S2, Table 2), although according to Ozçelik et al. [27] in Mueller Hinton broth a minimum inhibitory concentration of synephrine against *E. coli* ATCC 35218 was 8 μg/mL and *S. aureus* ATCC 25923—16 μg/mL.

The main bioactive compounds contained in *C. aurantium* are flavonoids (which are divided into flavanones, flavones and flavonols), limonoids (limonene and nomilin), alkaloids (*p*-synephrine), essential oils, coumarins, terpenoids, tannins, essential vitamins and minerals [48,49]. The list of bioactive compounds of *C. aurantium* differs somewhat for each part of the plant, as thoroughly described in the reviews by Maksoud et al. [48] and Fernández-Cabal et al. [49]. Since the manufacturer of the studied food supplement did not indicate which part of the plant was extracted, this complicates the analysis and comparison of results.

Maltodextrin, included in the formulation of the studied supplement **Cia**, is a carbon source for bacteria and is utilized as a targeting moiety for the precise localization of bacterial cells, while simultaneously reducing toxicity toward normal cells [50].

For **Yoh**, an aphrodisiac supplement used to manage erectile dysfunction, antibacterial activity was not established at a yohimbine concentration of 5 mg/mL using the antibacterial spot-on-lawn assay, as no zones of bacterial growth inhibition were observed (Figure 3, Table 1). A twofold decrease in the optical density of the bacterial cultures was observed upon diluting **Yoh** (30 and 300 μg yohimbine/mL medium) in the broth assay (Figure S2, Table 2). This contrasts with the findings of Özçelik et al. [27], who reported minimum inhibitory concentrations of 4 μg/mL against *E. coli* ATCC 35218 and 2 μg/mL against *S. aureus* ATCC 25923.

Such variation could be attributed to differences in the formulation of the studied preparations. The yohimbine-containing supplement analyzed in this study contains several auxiliary components, each characterized by distinct functional properties. Specifically, potato starch is used as a basis for creating films with antibacterial properties by incorporating antimicrobial compounds [51,52,53]. Lactose monohydrate acts as a carbon and energy source for bacterial growth, though, like starch, it exhibits no intrinsic antibacterial activity [54]. Calcium stearate functions as a stabilizer or thickener [55], while hypromellose is a water-soluble nonionic polymer with swelling and dissolution properties, and serves as a biocompatible matrix for drug delivery [56,57,58]. Cellactose provides superior tabletability and flowability [59].

In general, the antibacterial activity of yohimbine in vitro has been described in publications against a number of pathogenic microorganisms, both Gram-positive and Gram-negative bacteria. In particular, in addition to *E. coli* and *S. aureus*, it is able to inhibit the growth of other bacteria, including *Pseudomonas aeruginosa* [27,60]. The antibacterial activity of the crude extract of *Rauwolfia serpentina* root, which, among other alkaloids, contains yohimbine, was demonstrated using the antibacterial spot-on-lawn assay against *Salmonella typhi* bacteria (diameter of the zone of growth inhibition: 22.5 ± 2.5 mm) [61]. It has also been demonstrated that indole alkaloids (including α-yohimbine and its derivatives) can reduce the minimum inhibitory concentration of tetracycline against multidrug-resistant *E. coli* strains, which is associated with the ability of these compounds to inhibit bacterial efflux pumps [60]. However, it is noted that this effect is mainly synergistic and manifests itself in combination with antibiotics, while the antibacterial activity of the compounds themselves is limited [60].

To date, research has focused mainly on the metabolic and neuroendocrine effects of yohimbine, in particular on the regulation of oxidative stress and sympathetic activity [15,62]. However, given its antibacterial properties, further research should focus on the effects of dietary supplements with yohimbine on the intestinal microbiota. This is especially relevant, given the possibility of an indirect effect of yohimbine on microbial communities, which cannot be excluded, given the known interaction between the neuroendocrine system and the intestinal microbiota [63,64].

The observed differences in the antibacterial activity of turmeric, *C. aurantium* extract containing synephrine, and yohimbine between agar-based and liquid medium-based assay methods may be attributed to methodological factors, specifically the compound’s diffusion, solubility, and distribution within the test medium [65]. The antibacterial activity of plant extracts can be determined by the type of solvent used [66]. The solubility of curcumin in ethanol (96%) is approximately 29 times higher than in distilled water [67]. It is a lipophilic compound [68]. Lipophilicity is an important characteristic of compounds that exhibit antibacterial properties [69].

The presence of alkaloids (stachydrine, caffeine, cathine, synephrine, hordenine, cytisine, N-methylcytisine, and choline) in the ethanol extracts of *C. aurantium* [49] may explain the moderate antibacterial activity of the alcoholic solution of the commercial sports supplement **Cia** when tested on agar-based nutrient media. When water was used as a solvent, fatty acids were likely extracted [49], which reduced the antibacterial properties of the **Cia** solution in a liquid medium.

Yohimbine is highly soluble in alcohol but poorly soluble in water [70]. The absence of antibacterial activity on an agar medium could be attributed to an interaction with the solid medium [71], low diffusion [72], or partial inactivation during the diffusion process—a phenomenon described in the context of antibiotic diffusion in solid media [71].

Although the study demonstrated the antibacterial properties of turmeric- and synephrine-containing food supplements, these effects should not be directly attributed solely to the plant-based components, as the products contain various excipients that were not evaluated for antibacterial activity—a limitation of the present study. Furthermore, expanding the range of test strains—specifically by including antibiotic-resistant strains—would enhance the scientific value of the study, a factor that should certainly be taken into account in future research in this area. At the same time, the concentrations of curcumin, synephrine, and yohimbine tested for the antibacterial study lack clinical relevance. According to published data, the concentrations of these compounds reached in human blood plasma upon consumption are as follows: curcumin (10–12 g of native powder)—1.73 to 2.30 µg/mL [73]; synephrine (21 mg of *p*-synephrine)—1.75 ± 0.79 ng/mL (under resting conditions) and 1.86 ± 0.72 ng/mL (during physical exertion) [74]; and yohimbine (blood plasma/serum concentrations in man)—approximately 0.05–0.3 µg/mL for therapeutic (“normal”) levels, and 10 µg/mL for levels associated with coma or death [75].

## 4. Conclusions

Therefore, the results obtained demonstrate moderate antibacterial activity of the studied food supplements, primarily those containing turmeric and *C. aurantium* extract (with synephrine content), against some representatives of sanitary indicator bacteria—*Escherichia coli* and *Staphylococcus aureus*. These conclusions are consistent with published studies on the antibacterial properties of the active components—curcumin, demethoxycurcumin, bismethoxycurcumin, and *C. aurantium* extract (with synephrine). The less pronounced antibacterial effect of the food supplement with yohimbine may be associated with the content, in addition to the active component—yohimbine, of auxiliary compounds, in particular, starch and lactose can be a source of energy/carbon for microorganisms.

Given the results obtained, it is advisable to conduct further research on dietary supplements containing turmeric and synephrine to evaluate their antibacterial potential, which requires further study, particularly involving a broader range of test strains. Future studies should focus on specific molecular mechanisms of action, establishing effective concentrations, and assessing safety during long-term use. Particular attention should be paid to analyzing possible interactions with commonly used antibacterial drugs and assessing the risks of antibiotic resistance.

## Figures and Tables

**Table 1:** Antibacterial activity of the tested food supplements against bacterial test cultures (spot-on-lawn antibacterial assay).

Experiment Option	Growth Inhibition Zone Diameter, mm	
	*E. coli*	*S. aureus*
**Tur** (5 mg/mL)	18.0 ± 3.0 *	18.3 ± 0.3 *
**Cia** (5 mg/mL)	39.7 ± 2.6 *	18.0 ± 0.6 *
**Yoh** (5 mg/mL)	0	0
Ethanol (96%)	0	0
Tobramycin (10 μg/disk)	27.2 ± 0.7	35.0 ± 2.9

Note: **Tur**—with turmeric; **Cia** with *C. aurantium* extract containing synephrine; **Yoh** with yohimbine; * Differences from ethanol (solvent) are statistically significant at *p* ≤ 0.05.

**Table 2:** Optical density of bacterial cultures after 24-h cultivation in the presence of the tested food supplements.

Experiment Option	McF Values for the Used Test Cultures *	
	*E. coli*	*S. aureus*
Culture control	>4 ^a^	>4 ^a^
**Tur** (30 μg turmeric/mL medium)	1.1 ± 0.06 ^d^	1.9 ± 0.07 ^c^
**Tur** (300 μg turmeric/mL medium)	1.0 ± 0.09 ^d^	1.3 ± 0.1 ^d^
**Cia** (30 μg synephrine/mL medium)	2.3 ± 0.4 ^b^	>4 ^a^
**Cia** (300 μg synephrine /mL medium)	>4 ^a^	>4 ^a^
**Yoh** (30 μg yohimbine/mL medium)	1.8 ± 0.2 ^c^	2.5 ± 0.2 ^b^
**Yoh** (300 μg yohimbine/mL medium)	1.3 ± 0.07 ^d^	2.2 ± 0.1 ^b^
Ethanol (5.8% in medium)	>4 ^a^	>4 ^a^
Tobrex (30 μg tobramycin/mL medium)	0.59 ± 0.01 ^e^	0.59 ± 0.01 ^e^
Tobrex (300 μg tobramycin/mL medium)	0.56 ± 0.01 ^e^	0.55 ± 0.01 ^e^

**Note:** *—The results are presented as the difference between the turbidity values for the “bacteria + dietary supplement + medium” condition and the “dietary supplement + medium” control. **Tur**—with turmeric; **Cia** with *C. aurantium* extract containing synephrine; **Yoh** with yohimbine; Different superscript letters indicate statistically significant differences between groups according to the post hoc test Tukey’s HSD (*p* < 0.05).

## Data Availability

The original contributions presented in this study are included in the article/Supplementary Materials. Further inquiries can be directed to the corresponding author.

## Supplementary Materials

The following supporting information can be downloaded at https://www.mdpi.com/article/10.3390/microorganisms14102399/s1: Figure S1: Controls used in studies of the antibacterial effect of food supplements (a spot-on-lawn antibacterial assay); Figure S2: Antibacterial effect of the food supplements (method with a liquid nutrient medium): (**a**)—*E. coli*; (**b**)—*S. aureus*; Figure S3: Sterility controls of alcoholic solutions of dietary supplements used in studies of their antibacterial action (method with a liquid nutrient medium).

## Abbreviations

The following abbreviations are used in this manuscript:

*E. coli*	*Escherichia coli*
*S. aureus*	*Staphylococcus aureus*
Cia	The food supplement with *Citrus aurantium* extract (with synephrine)
Tur	The food supplement with turmeric
Yoh	The food supplement with yohimbine
