# Supplementary material for: Evaluation of the Antibacterial Properties of Turmeric-, Synephrine- and Yohimbine-Containing Dietary Supplements Used by Bodybuilders
Source: Microorganisms. 2026 Oct 20;14(10):2399. doi: 10.3390/microorganisms14102399 (PMC13707916; doi:10.3390/microorganisms14102399)
Supplement: Supplementary file 1 [file microorganisms-14-02399-s001.zip › microorganisms-4636698-supplementary.pdf]

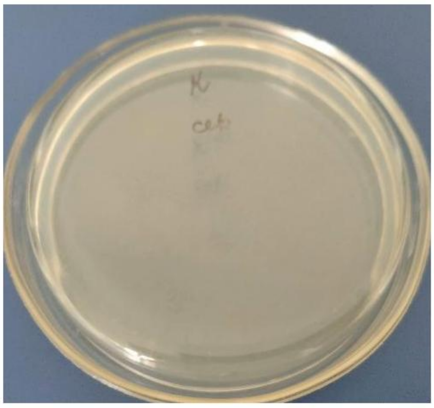

Sterility control

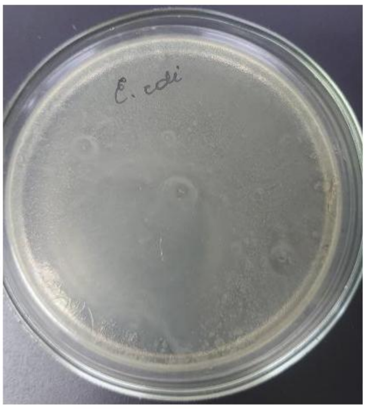

Culture control (*E. coli*)

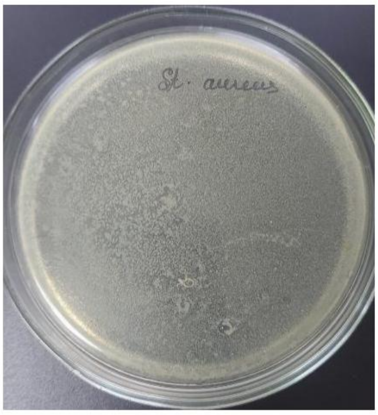

Culture control (*S. aureus*)

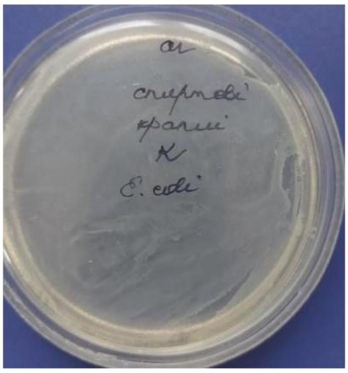

Solvent control – ethyl alcohol (*E. coli*)

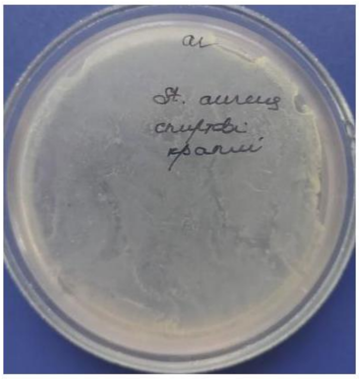

Solvent control – ethyl alcohol (*S. aureus*)

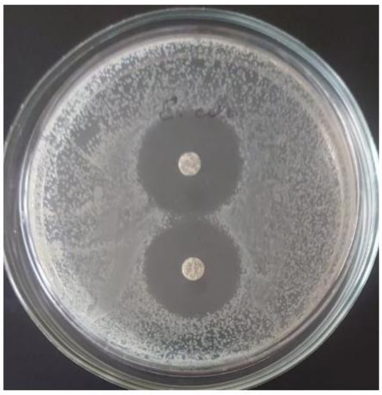

Positive control – antibiotic tobramycin (*E. coli*)

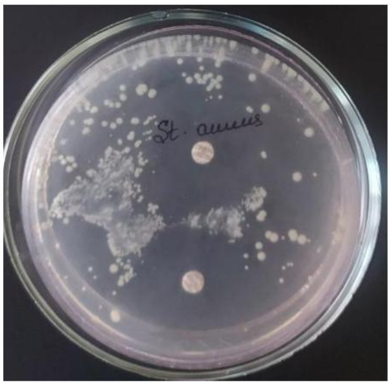

Positive control – antibiotic tobramycin (*S. aureus*)

Figure S1. Controls used in studies of the antibacterial effect of food supplements (a spot-on-lawn antibacterial assay)

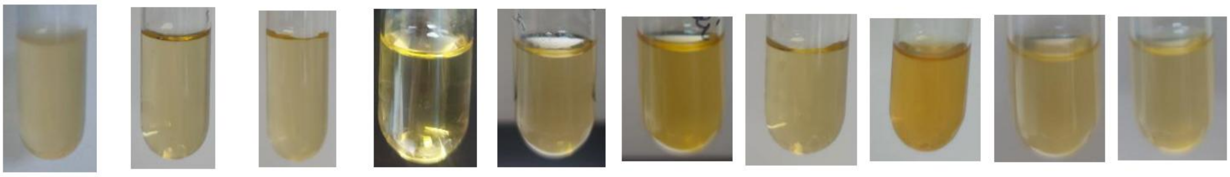

Culture control	Control of sterility of the medium	Solvent control (ethyl alcohol)	Positive control (Tobrex, 30 μg antibiotic tobramycin/ml medium)	30 μg turmeric/ml medium	300 μg turmeric/ml medium	30 μg synephrine/ml medium	300 μg synephrine/ml medium	30 μg yohimbine/ml medium	300 μg yohimbine/ml medium

(a)

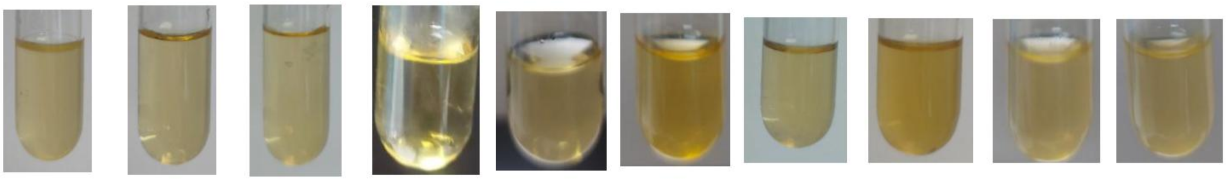

Culture control	Control of sterility of the medium	Solvent control (ethyl alcohol)	Positive control (Tobrex, 30 μg antibiotic tobramycin/ml medium)	30 μg turmeric/ml medium	300 μg turmeric/ml medium	30 μg synephrine/ml medium	300 μg synephrine/ml medium	30 μg yohimbine/ml medium	300 μg yohimbine/ml medium

(b)

Figure S2. Antibacterial effect of the food supplements (method with a liquid nutrient medium): (**a**)—*E. coli*; (**b**)—*S. aureus*

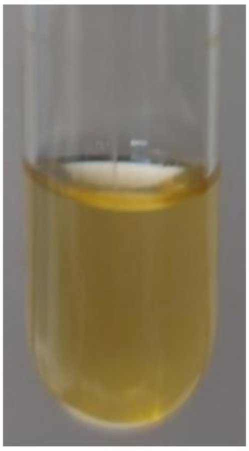

Tur (30 μg turmeric/ml medium)

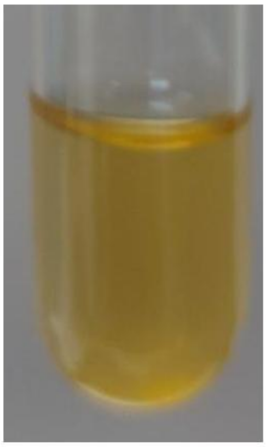

Tur (300 μg turmeric/ml medium)

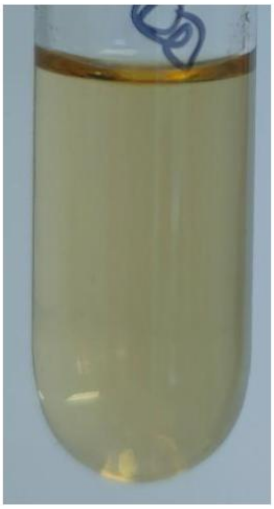

Cia (30 μg synephrine/ml medium)

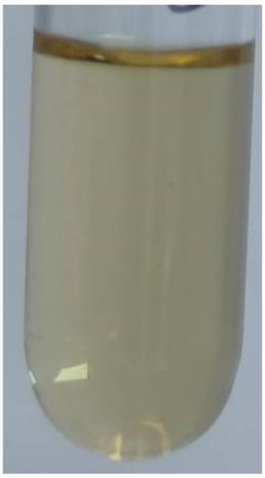

Cia (300 μg synephrine/ml medium)

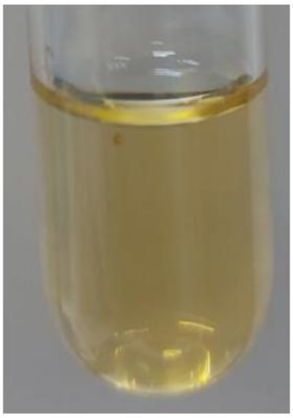

Yoh (30 μg yohimbine/ml medium)

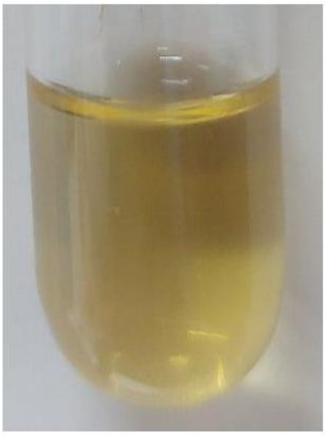

Yoh (300 μg yohimbine/ml medium)

Figure S3. Sterility controls of alcoholic solutions of dietary supplements used in studies of their antibacterial action (method with a liquid nutrient medium)
